# Harnessing fungal endophytes for natural management: a biocontrol perspective

**DOI:** 10.3389/fmicb.2023.1280258

**Published:** 2023-12-08

**Authors:** Mansavi Bhardwaj, Swadha Kailoo, Rabiya Tabbassum Khan, Sofia Sharief Khan, Shafaq Rasool

**Affiliations:** Molecular Biology Laboratory, School of Biotechnology, Shri Mata Vaishno Devi University, Katra, Jammu and Kashmir, India

**Keywords:** fungal endophyte, biocontrol mechanism, secondary metabolites, sustainable agriculture, bioactive potential

## Abstract

In the ever-evolving realm of agriculture, the convoluted interaction between plants and microorganisms have assumed paramount significance. Fungal endophytes, once perceived as mere bystanders within plant tissues, have now emerged as dynamic defenders of plant health. This comprehensive review delves into the captivating world of fungal endophytes and their multifaceted biocontrol mechanisms. Exploring their unique ability to coexist with their plant hosts, fungal endophytes have unlocked a treasure trove of biological weaponry to fend off pathogens and enhance plant resilience. From the synthesis of bioactive secondary metabolites to intricate signaling pathways these silent allies are masters of biological warfare. The world of fungal endophytes is quite fascinating as they engage in a delicate dance with the plant immune system, orchestrating a symphony of defense that challenges traditional notions of plant-pathogen interactions. The journey through the various mechanisms employed by these enigmatic endophytes to combat diseases, will lead to revelational understanding of sustainable agriculture. The review delves into cutting-edge research and promising prospects, shedding light on how fungal endophytes hold the key to biocontrol and the reduction of chemical inputs in agriculture. Their ecological significance, potential for bioprospecting and avenues for future research are also explored. This exploration of the biocontrol mechanisms of fungal endophytes promise not only to enrich our comprehension of plant-microbe relationships but also, to shape the future of sustainable and ecofriendly agricultural practices. In this intricate web of life, fungal endophytes are indeed the unsung heroes, silently guarding our crops and illuminating a path towards a greener, healthier tomorrow.

## 1 Introduction

**Figure 1:**
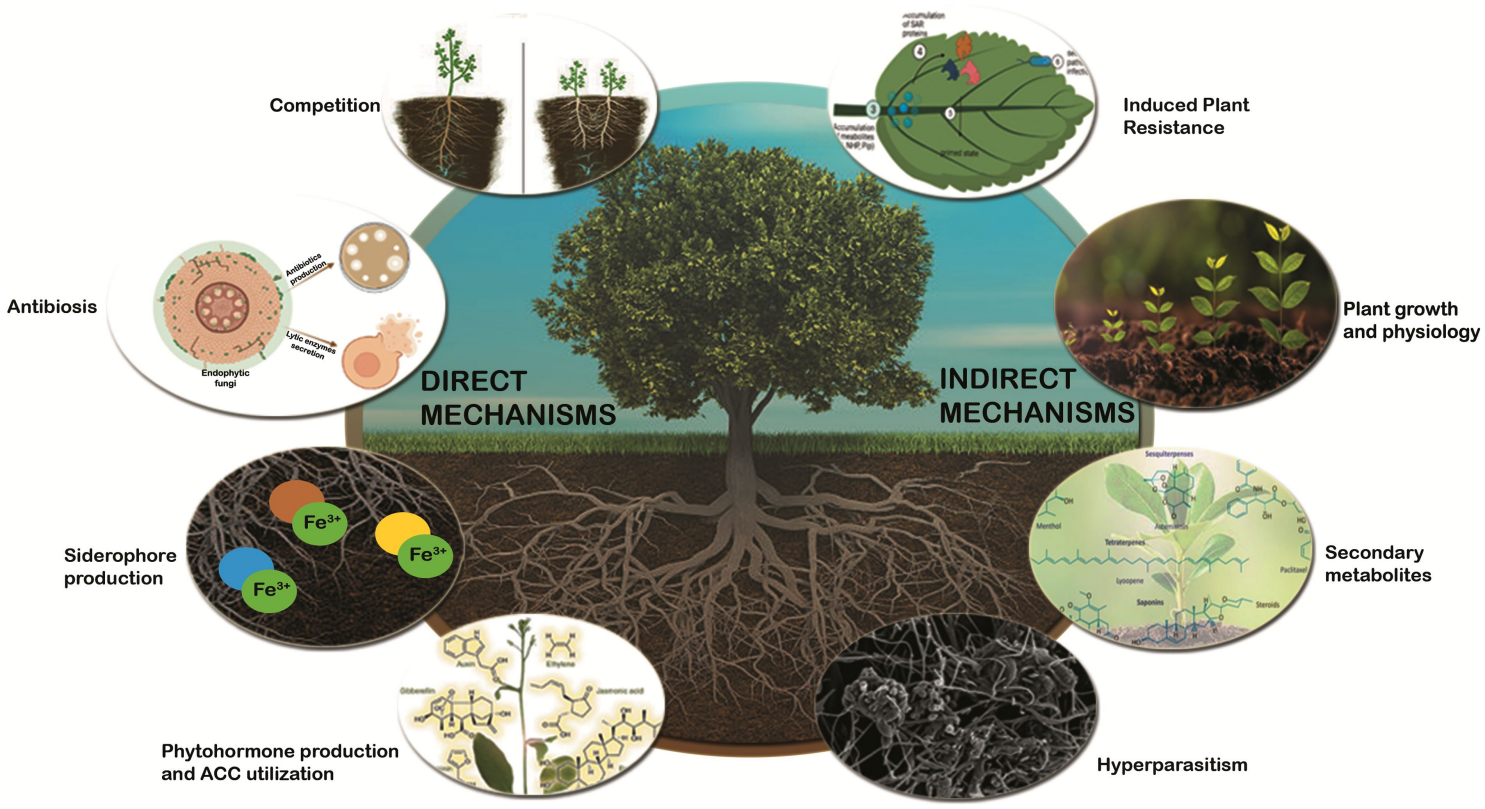
Schematic representation of defense mechanisms used by endophytic fungi for plant protection against phytopathogens.

In the realm of agriculture and plant health management, the quest for sustainable and environmentally friendly alternatives to conventional methods has never been more pressing. The exponential growth of the global population, along with the ever-escalating demands for food production, places immense pressure on our agricultural systems. In this view, the health of cultivated plants is crucial for many economic sectors because plants not only offer food for the inhabitants, but also critical items like wood, textiles, medicines, and bioenergy, among others. Plant diseases are to blame for production losses that are both large in terms of quantity and quality, costing businesses a lot of money, and occasionally having disastrous social effects (Savary et al., 2019). On a global scale, plant illnesses can result in losses of up to 16%, and studies have already shown that pathogens and, more precisely, conducted cultivations are the targets of losses (Savary et al., 2019). Without a doubt, diseases have the capacity to cause losses, and the severity of those losses may vary depending on the climate, microbes, and the aggressiveness of the disease-causing agent. For ages, numerous methods have been exploited to increase food production, many of which are unsustainable since they pose risks to the environment (Sahu and Mishra, 2021). According to this assumption, reducing these eco-threats and investigating potential endophytic microbes will aid in achieving an ecosystem that is stable and will enable the cultivation of pathogen-free plants for increased crop output. In this context, endophytic fungi have emerged as an intriguing and promising class of microorganisms that hold the potential to revolutionize modern agriculture (Sharma and Singh, 2021). In nature, both plants and microbes live in relationships among themselves, which in turn can affect the overall growth and development of plants. Endophytic microbes are an intriguing collection of organisms that are linked to diverse parts and tissues of plants. The association of these microbes with plants can be facultative or obligate and causes no harm to their host. On the other hand, these endophytes exhibit intricate relationships with their host plants, including antagonism and mutualism (Parker, 1995). One such widely acknowledged interaction of mutualism or symbiosis involves the interaction of medicinal plants and their associated endophytes. These endophytes are known to produce certain secondary metabolites of immense pharmacological importance (Elgorban et al., 2019). Fungal endophytes have gained a lot of attention from taxonomists, mycologists, ecologists, chemists, and evolutionary biologists over the last three decades (Saikkonen, 2007). They actually represent a diverse array of fungi that establish mutualistic relationships within the tissues of plants, all while evading the conventional signs of diseases or distress that are typically associated with pathogenic fungi. This intriguing class of microorganisms has remained hidden allies of the plant kingdom for centuries performing their roles quietly and unobtrusively. However, recent decades have witnessed a surge of research interest in these cryptic microorganisms, revealing their profound influence on plant growth, development, and resistance to abiotic and biotic stresses. The remarkable attribute of these fungi lies in their ability to form mutualistic associations with a wide range of plant species without causing overt symptoms of disease, setting them apart from their pathogenic counterparts (Saikkonen, 2007). Approximately 5% of the estimated million fungal species on earth have been described, which is a very tiny percentage (Panda and Sujogya., 2013). They are being studied due to their ability to boost plant health; for example, in *Musa* spp., *Piriformospora indica* has been found to induce resistance against *Fusarium oxysporium* f. sp. *cubense* (Cheng et al., 2020). These endophytes can affect the host plant’s biochemistry, physiology, distribution, and ecology, and their persistence has been known for the past 100 years. The effect of the association of endophytic fungi, on plant growth and immunity against pathogens greatly depends on the site of the colonization and the secondary metabolites produced by the endophytes (Yu et al., 2018). Several studies have shown that the presence of microbes in the plant endosphere affects the plant’s response to environmental stimuli and can regulate plant diseases (Zhang et al., 2020). Reports have shown that endophytic fungi have the potential to maintain plant health and can be engineered to integrate into crop breeding (Liu et al., 2018). An interesting fact about Rhizospheric microbes/fungi living in the proximity of root endosphere is that they can modify their morphology rapidly and can colonize the plant tissues, thus becoming endophytic microbe/ fungi (Abedinzadeh et al., 2019). It is interesting to note that data has demonstrated how endophytic fungi can colonize and infiltrate internal plant tissue (endosphere) from their external root environment (rhizosphere), establishing endophytic microbial communities (Vishwas, 2011). When compared to non-symbiotic plants, endophytes can strongly and quickly stimulate and activate the host plant’s stress response (Wu et al., 2018). As already stated above, plant pathogens have always been the first threat to food security in our world, and in most cases, the available tools were insufficient to effectively manage them. *Phytophthora infestans*, the first ever plant pathogen reported in tomato and other related cultivars, is still responsible for their reduced production. A lot of different hosts and most strains make it harder to use non-host crops and resistant cultivars. The use of chemically synthesized. Pesticides is not always that effective or applicable, both in terms of cost and method of application. On the other hand, security is expected not only in terms of food production but also in quality and overall impact on the environment (Lechenet et al., 2017). Plants can support a microbial community in the rhizosphere and even recruit some in adverse conditions. A fresh perspective on biological control, particularly through endophytes, offers innovative biotechnological methods of managing plant diseases and a unique viewpoint on the relationship between microbes and plants (Tena, 2018). As biocontrol agents, endophytic fungi, during their entire life cycle, protect the host plants from infections as they easily adjust to adverse environmental conditions (Xie et al., 2018). Interaction between endophytes and their host plants improves plant growth and protects the plant from harmful effects, e.g., by helping in developing resistance against pathogens, aiding in resistance mechanisms like phytoremediation, increasing the crop yield, etc. (Saikkonen et al., 2004). In this interaction, both endophytes and hosts benefit as the plant provides protection, nutrition, and shelter to them. Endophytes, on the other hand, assist their hosts by stimulating their growth, development, and adaptation (Killham, 1994). When the plant gets attacked by a disease pathogen, the host defense system gets activated, and protection against disease occurs mainly by minimizing the level of infection as well as masking and reducing the pathogen’s growth (Jia et al., 2016). Endophytes have helped their host plants by enhancing their growth and developing resistance against pathogens in the course of co-existence and evolution. This results in the establishment of special interactions such as mutualism, neutralism, and antagonism between the host plant and its endophytes (Jia et al., 2016). Due to their efficacy as biocontrol agents against several plant infections, the screening of endophytic fungi have recently increased, as reported (Abaya et al., 2021). Endophytic fungi that live in different plant compartments generally encourage plant growth in a variety of direct and indirect ways (Adeleke et al., 2022). In the direct mechanism, endophytes control different plant hormones such as auxins and cytokinins and improve the availability of soil nutrients through nitrogen fixation, siderophore production, phosphorus, and iron solubilization, whereas in the indirect mechanism, endophytes produce various volatile compounds like hydrogen cyanide, enzymes, and antibiotics that cease pathogen activity and promote systemic resistance in plants (Selim et al., 2018). It has been suggested that endophytic ecological occupation and phytoalexin synthesis caused by fungal endophytes may be the primary mechanisms by which plants protect themselves from diseases. This review paper embarks on an exploration of the multifaceted world of endophytic fungi, focusing on their extraordinary biocontrol mechanisms and their potential applications in sustainable agriculture. As the global community seeks innovative solutions to address challenges such as crop diseases, pests, and the adverse effects of climate change, endophytic fungi stand as nature’s hidden allies, offering a treasure trove of mechanisms that can be harnessed to combat these pressing issues. As one traverse the complex landscape of endophytic fungi and their biocontrol mechanisms, it becomes evident that these enigmatic organisms hold the key to a sustainable and resilient agricultural future. This review aims to spark more research and new ideas by shedding light on their many different strategies and the environmental factors that make them successful. This will help us learn more about these hidden allies and how important they are to global food security (Figure 1).

## 2 Direct mechanism against plant pathogens

Endophytes have been the subject of recent research, which has shown that they can improve host defense against diseases and lessen the ecological harm that pathogenic microorganisms can cause (Arnold et al., 2003). Most of these investigations involved the assessment of the survival rate of plants in the presence and absence of fungal endophytes or *in vitro* coculture of fungal endophytes and phytopathogens. Although some studies suggest that endophytes may reduce the effects of plant-pathogen damage, the current understanding of the precise regulation of endophytes, pathogens, and plants is still in the beginning stages (Mejía et al., 2008). The direct mechanism employed by endophytes for the protection of plants against phytopathogens involves direct antagonistic measures such as antibiosis, competitive exclusion of phytopathogens, parasitism and elucidation of pathogen virulence (Köhl et al., 2019). Endophytes can exert antibiosis by secreting allelochemicals like bacteriocins, lipopeptides, biosurfactants, enzymes that break down cell walls, antibiotics, and volatile chemicals that interfere with phytopathogen metabolism and hence stop pathogen development (Raymaekers et al., 2020). Additionally, competition between the endophytes and the pathogens for nutrition and space also plays a role in the reduction of pathogenic infection in plants. Secretion of enzymes like pectinases and chitinases also inhibits the virulence of pathogen by interfering with factors responsible for pathogenicity in phytopathogens (Wang et al., 2022). However, the direct interactions between endophytic fungi and phytopathogens are complicated and species-specific in nature (Gouda et al., 2016).

### 2.1 Antibiotics production from endophytes

Studies throughout the decades have shown that endophytes are central to plant protection against pathogens (Barkodia et al., 2018). During the direct interaction between endophytes and pathogens, endophytes produce antibiotics to suppress the pathogens and protect the plant from the damage caused by pathogens (Fadiji and Babalola, 2020). Endophytes produce certain secondary metabolites that have antimicrobial properties like antifungal, antibacterial, etc. (Palanichamy et al., 2018). These antimicrobials have a strong inhibitory action on plant pathogens. A single class of endophytes is capable of producing a variety of bioactive agents (antibiotics) such as aromatic compounds, terpenoids, polypeptides, and terpenoids (Balla et al., 2021). Antimicrobials synthesized by some endophytic fungi are listed in Table 1.

**Table 1:** List of antimicrobials produced by endophytic fungi for plant protection.

S. no.	Endophyte	Antimicrobial compound	Plant pathogen inhibited	Reference
1	*Acremonium zeae*	Pyrrocidines A, Pyrrocidines B	*Fusarium verticillioides, Aspergillus flavus*	Wicklow and Poling (2008)
2	*Ampelomyces* spp.	3-O-Methylalaternin, Altersolanol A	*Staphylococcus aureus, Enterococcus faecalis* and *S. epidermidis*	Wen et al. (2023)
3	*Cladosporium* spp.	Brefeldin A	*Trichophyton, Candida albicans,* and *Aspergillus niger*	Akram et al. (2023)
4	*Colletotrichum gloeosporioides*	Methanol	*Escherichia coli, Pseudomonas aeruginosa*	Wang et al. (2021)
5	*Daldinia concentrica*	Volatile organic compounds	*Aspergillus niger, Penicillium digitatum, and Botrytis cinerea*	Lugtenberg et al. (2016)
6	*Gliocladium* spp.	Volatile organic compounds like 1- butanol, 3-methyl-, phenylethyl alcohol and acetic acid, 2-phenylethyl ester,	*Pythium ultimum* and *Verticillum dahlia*	Lugtenberg et al. (2016)
7	*Muscodor albus*	Aciphyllene, Tetrohydofuran, 2- butanone, 2-methyl furan	*Stachybotrys chartarum*	Saxena et al. (2015)
8	*Phomopiscassiae*	Cadinane sesquiterpenes derivatives	*Cladosporium sphaerospermum* and *Cladosporium cladsporioides*	Gao et al. (2010)
9	*Periconia* spp.	Fusicoccane diterpenes	*Salmonella typhimurium, Staphylococcus aureus, Klebsiella pneumoniae,* and *Bacillus subtilis,*	Azhari and Supratman (2021)
10	*Paraconiothyrium* spp.	Taxol/ Paclitaxel	*Heterobasidion annosum, Phaeolus schweinitzii and Perenniporia subacida*	Talbot (2015)
11	*Pestalotiopsis microspora*	Torreyanic acid	*---*	Ding et al. (2009)
12	*Penicillium* spp.	Penicitroamide	*Erwinia carotovora sub* sp. *Carotovora*	Deshmukh et al. (2022)
13	*Trichoderma* spp.	Trichodecenins, Trichorovins, Trichocellins, Trichorzianins A and B, Trichorzins, HA and MA, Tricholongins BI and BII Longibrachinsa	*Rhizoctonia solani, Botrytis cinerea, Phytophthora cinnamomi, Pythium irregulare, Pythium middletonii, Sclerotinia sclerotiorum, Fusarium oxysporum, Bipolaris sorokiniana*	Sood et al. (2020)
14	*Verticillium* spp.	Massariphenone ergosterol peroxide	*Pyricularia oryzae* P-2b	Rani et al. (2017)

### 2.2 Secretion of lytic enzymes

Lytic enzyme secretions play a significant role in plant protection from pathogens, particularly when it comes to endophytic microorganisms. Most microbes produce lytic enzymes that aid in the hydrolysis of polymers (Tripathi et al., 2008). Endophytic fungi create a symbiotic relationship with plants when they colonize the internal tissues of plants; this interaction is advantageous to both the plant and the fungus. As pathogens attempt to invade the plant, the fungal endophytes parasitize pathogenic hyphae in various ways, including twisting, coiling, perforation of the pathogenic hyphae, and secretion of cell wall-degrading enzymes leading to lysis and inhibition of pathogens (Waghunde et al., 2017). By degrading the cell walls of pathogens, the lytic enzymes limit the ability of the pathogens to invade and propagate within the plant, effectively enhancing the plant’s natural defense mechanisms. This mechanism not only provides immediate protection but also primes the plant’s immune system for induced systemic resistance (ISR), leading to long-lasting and robust defense responses against potential future pathogen attacks (Fadiji and Babalola, 2020; Trivedi et al., 2020). In lieu of chemical pesticides, the use of lytic enzymes produced by endophytic fungi offers a sustainable and environmentally benign method of plant protection, encouraging healthier and more resilient crops. Despite their involvement in mycoparasitism, these lytic enzymes also contribute to cell wall reformation and recycling during active fungal growth, as well as during aging and autolysis (Kumari and Srividhya, 2020). Endophytic fungi have a variety of enzymes such as hemicellulases,1,3-glucanases, chitinases and cellulases, that break down different types of materials (Haran et al., 1996). It has been reported that in *Trichoderma* species, a number of enzymes are directly involved in the breakdown of the cell wall of pathogenic fungi for the utilization of pathogenic fragments (Rajesh et al., 2016). Mycolytic enzymes like 1,3-glucanase or chitinase produced by *Trichoderma asperellum* have the potential to degrade the cell walls of phytopathogens. A respective increase in enzymatic activity and transcripts of 1,3, glucanase, and chitinase enzymes in cultures induced by pathogens has been observed in banana wilt affected plantations (Win et al., 2021). Biocontrol of root-knot nematodes by endophytic fungi showed elevated activity of genes encoding enzymes like glucanase and chitinase and downregulation of genes encoding antioxidant enzymes during the stimulation of the immune system of plants in response to pathogens (Molinari and Leonetti, 2019). Although enzymes lack the ability to act as antagonizing agents independently, but they augment the antagonistic activity of other agents and pathways when merged together. Similarly, the pectinase enzyme has also been reported to help reduce pathogenesis in plants (Fadiji and Babalola, 2020).

### 2.3 Production of phytohormones

**Figure 2:**
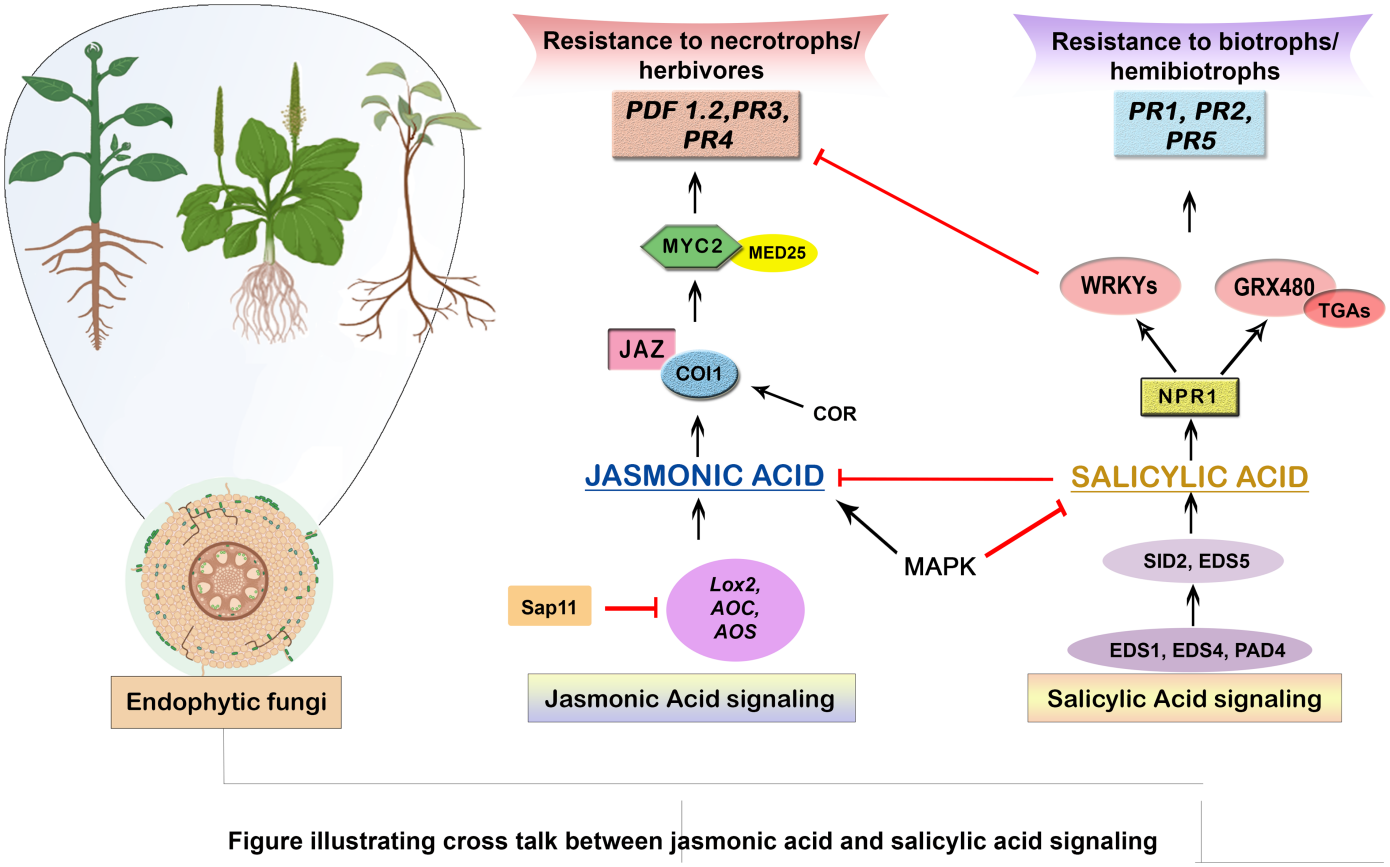
Illustrating cross talk between Jasmonic acid and Salicylic acid signaling. This schematic diagram depicts the intricate interplay between two key plant defense signaling pathways, Jasmonic acid (JA) and Salicylic acid (SA), in response to various biotic and abiotic stressors. The crosstalk between these signaling pathways plays a pivotal role in shaping a plant’s response to different types of threats.

Phytohormones of endophytic fungi play a crucial role in plant protection, enhancing defense responses and resistance to pathogens through complex interactions with the plant (Fadiji and Babalola, 2020). The context of plant protection, the secretion of phytohormones by endophytic fungi trigger a sequence of defense mechanisms in the host plant. In response to environmental cues, endophytic fungi that have formed a symbiotic relationship with plants release phytohormones such as auxins, cytokinins, gibberellins, indole acetic acid, and jasmonic acid that act as potent signaling molecules (Baron and Rigobelo, 2022). The two phytohormones, Jasmonic acid (JA) and Salicylic acid (SA) are important defense signaling molecules that regulate the defense responses of plants against disease-causing microbes (Shi et al., 2020). Systemic acquired resistance (SAR) and biotrophic pathogen defense are both impacted by Salicylic acid, which in turn is regulated by the activation of Pathogenesis-related (PR) genes. Localized programmed cell death, a fallout of the hypersensitive response caused by the two phytohormones, jasmonic acid and salicylic acid inhibits the spread of pathogens and protects against stress (Lahlali et al., 2022). These proteins and compounds act as an immediate response to pathogen attack, hindering the progress of invading pathogens and limiting their growth (Rashad et al., 2020). When pattern recognition receptors (PRRs) recognize the pathogens, Jasmonic acid (JA) and Salicylic acid (SA) have the potential to enhance the activity of the enzymes in the phenylpropane pathway of plants resulting in the production of phenolic compounds (such as flavonoids, lignin, coumarins, and tannins), triggering pathogen-associated molecular pattern (PAMP)-triggered immunity (PTI), a robust defense response in plants (Franco-Orozco et al., 2017). Jasmonic acid and ethylene (ET), mainly produced during induced systemic resistance (ISR), are considered important for defense against necrotrophic diseases and beneficial for interactions between plants and pathogens (Li et al., 2019). The phenomenon of JA/ET-dependent systemic resistance has been seen in various plant-associated microorganisms, including Trichoderma asperellum, Penicillium sp., and the endophyte *Serendipita indica*. But in other pathosystems, *S. indica* produced resistance without relying on the JA/ET route, while *T. asperellum* coupled with plants triggered resistance in an SA-dependent manner. This suggests that the roles of phytohormones and their potential interactions are complicated, and the use of a microorganism on the plant is expected to alter the entire hormone profile rather than solely altering the levels of individual hormones. Plant defense signaling pathways have been identified and compounds act as an immediate response to pathogen attack, hindering the progress of invading pathogens and limiting their growth (Rashad et al., 2020). When pattern recognition receptors (PRRs) recognize the pathogens, jasmonic acid (JA) and salicylic acid (SA) have the potential to enhance the activity of the enzymes in the phenylpropane pathway of plants, resulting in the production of phenolic compounds (such as flavonoids, lignin, coumarins, and tannins), triggering pathogen-associated molecular pattern (PAMP)-triggered immunity (PTI), a robust defense response in plants (Bhattacharya et al., 2010). Jasmonic acid and ethylene (ET), mainly produced during induced systemic resistance (ISR), are considered important for defense against necrotrophic diseases and beneficial for interactions between plants and pathogens (Ghozlan et al., 2020). Plant defense signaling pathways have been found to involve ethylene (ET) and abscisic acid (ABA), while auxin, gibberellic acid (GA), cytokinin (CK), brassinosteroids, and peptide hormones may also be involved (Li et al., 2019). Furthermore, ethylene and jasmonic acid also initiate the synthesis of secondary metabolites, including volatile organic compounds (VOCs) and phytoalexins. VOCs can serve as signaling molecules to attract beneficial microorganisms, such as predatory insects or microbes that feed on plant pests, to further enhance plant protection. On the other hand, phytoalexins are antimicrobial compounds that are directly involved in the inhibition of pathogen growth and proliferation within the tissues of plants (Latz et al., 2018). A study conductedal on the functional role of *Trichoderma atroviride* fungi in controlling the pathogenic activity of *Fusarium verticillioides* in maize showed the involvement of this endophytic fungi in the synthesis of phytohormones such as salicylic acid, abscisic acid (ABA) and jasmonic acid (Agostini et al., 2019). Similarly, Ren and Dai also found that the interaction between *Gilmaniella* spp. and endophytic fungi *Atractylodes lancea* led to increased production of jasmonic acid along -with the production of various volatile antimicrobial compounds such as eudesmol, atractylone, atractylodin and hinesol (Ren and Dai, 2012). Together, ethylene, Jasmonic acid, and salicylic acid create a hormonal response network that is coherent and keeps the plant’s defense mechanisms in place like production of compounds like ethylene and jasmonic acid in response to agents like *Penicillium* spp., *Serendipita indica* and *Trichoderma asperellum* to activate systemic resistance is crucial in avoiding host-pathogen inhabitation or activation of salicylic acid dependent systemic resistance pathway in plants inhabited by *Trichoderma asperellum* (Latz et al., 2018). These hormonal responses generated between the endophytic fungi and phytopathogens are extremely complicated and involves cross communication and multiple events between plants and the host endophytic fungi (Adeleke et al., 2022; Figure 2).

**Figure 3:**
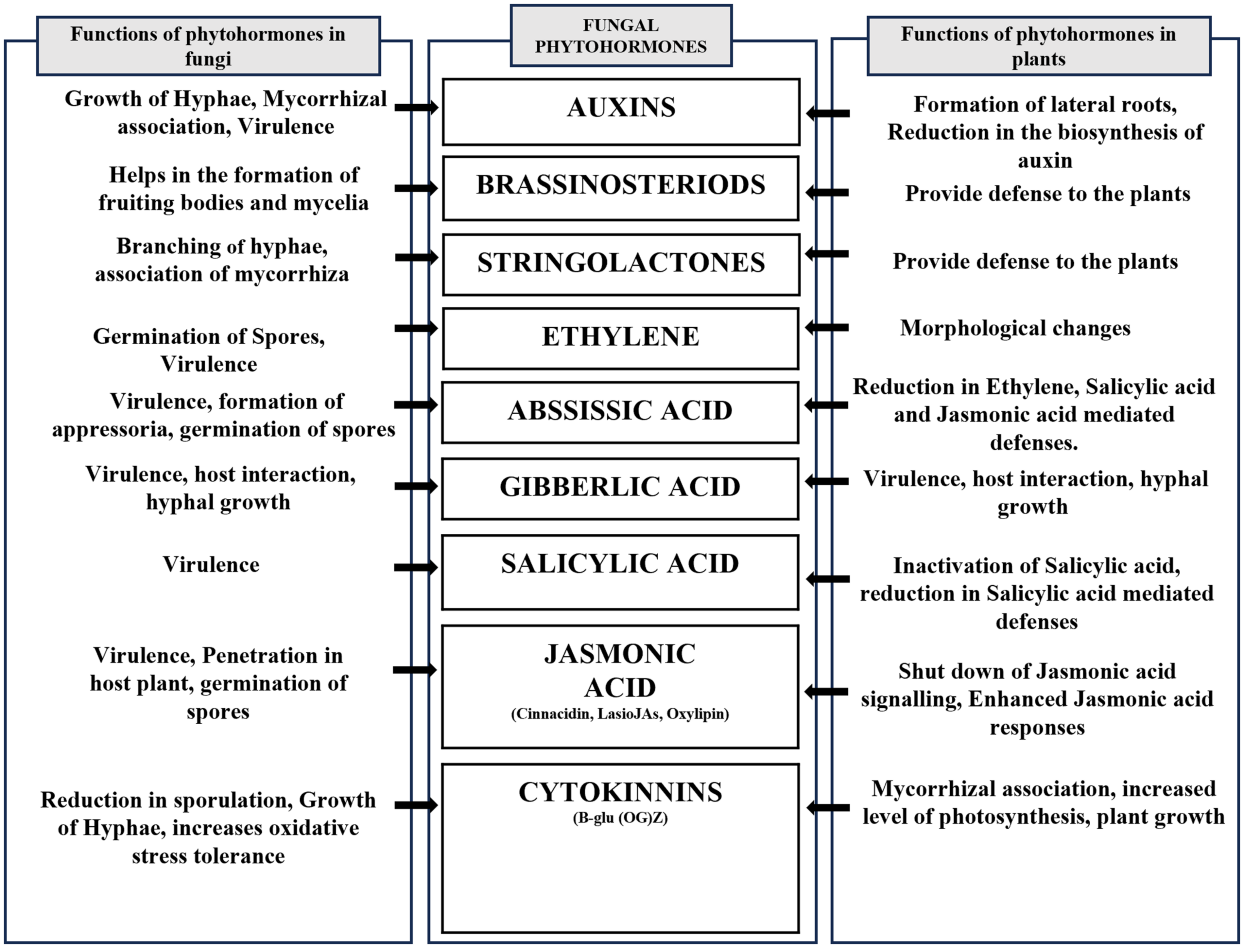
Illustrating phytohormones produced by endophytic fungi. This schematic diagram depicts different phytohormones produced by endophytic fungi and their role both in endophytic fungi and their host.

Moreover, phytohormones influence the establishment of induced systemic resistance (ISR) in the plant. Induced Systemic Resistance entails getting the plant’s immune system ready to react more quickly to future pathogen attacks. This priming effect allows the plant to mount a quicker and more robust defense, effectively protecting it from potential pathogen threats (Baron and Rigobelo, 2022). Despite significant advances in research on signal transmission in induced resistance, there is still a lacuna in allocating roles to each hormone in signal transduction, particularly in complex systems. As a result, there is a need to strengthen a plant’s defense systems and use it as a biomarker to detect induced resistance (Latz et al., 2018). Overall, the secretion of phytohormones by endophytic fungi in plant protection is a finely tuned mechanism that involves a network of signaling pathways and defense responses. By modulating the plant’s hormonal balance, endophytic fungi contribute to a heightened state of preparedness against pathogen attacks, leading to improved plant health, resilience, and reduced reliance on chemical pesticides. Harnessing the potential of plant phytohormones from endophytic fungi offers a sustainable and eco-friendly approach to plant protection, benefiting both agricultural productivity and environmental health (Waghunde et al., 2017; Figure 3).

#### 2.3.1 Volatile and non-volatile compounds of fungal endophytes

The metabolites produced by the endophytic fungi are primarily of two kinds: Volatile compounds and Non-Volatile Compounds (Santra and Banerjee, 2023). Volatile compounds can be defined as low molecular weight hydrophobic organic molecules with a high vapor pressure. Volatile organic compounds (VOC) are the name for the volatile metabolites that endophytic fungi produce. These volatile compounds can easily cross the cell membrane of the plant thus playing a vital role in soil ecosystem. The majority of VOC can be categorized into five different classes namely: Benzenoid compounds, Terpenoids, Amino acid derivatives, Fatty acid derivatives and Phenylpropanoids. These are mostly antibiotic in nature (Kaddes et al., 2019). The non-volatile metabolites produced by endophytic fungi comprise a wide range of chemically different compounds such as peptides, polyketides, steroids, enzymes, alkaloids, amino acids, hormones etc. (Singh and Kumar, 2023). Both VOC and non-volatile metabolites have a wide role in agriculture and help in plant protection against biotic and abiotic stress (Singh and Kumar, 2023). Some of the examples are shown below in Table 2.

**Table 2:** List of volatile and non-volatile compounds produced by fungal endophytes and their role in plant protection.

Type of metabolite	Metabolite	Examples	Producer endophyte	Activity of the metabolite	Reference
	Terpenes	Azadirachtin A & B, Camptothecin, Penicimonoterpene, Sabinene, Geraniol	*Eupenicillium parvum; Fusarium solani, Penicillium chrysogenum Phomopsis* spp., *C. elatior Sw.*	Biopesticide, Antifungal	Kilani-Morakchi et al. (2021)
Volatile organic compounds	Phenols	Phenol, 4-[2-(methylamino) ethyl]-, 6-Nitro-3-chlorophenol, Phenol, 2,4,6-tri-tert-butyl-	*Curvularia* spp.	Antifungal	Santra and Banerjee (2023)
	Fatty acid derivatives	1-octen-3-ol and 3-octanone	*Muscodor* spp.	Fungicides, Pesticides, Phytotoxicity, Insecticides	Plaszkó et al. (2020)
	Alcohol	1-butanol, phenethyl alcohol, 1- propanol, isobutyl alcohol, 2- methyl-1-butanol, cyclohex-3-en-1- ol	*Phomopsis* spp. *Muscudor* CZ-620	Antifungal	Kaddes et al. (2019)
	Ketone	Acetone	*Phomopsis* spp.	Antifungal	Kaddes et al. (2019)
	Amino-acid derivative	Bactobolin & Actinobolin	*C. elatior Sw.*	Antifungal/defe nse against phytopathogen	Santra and Banerjee (2023)
	Seco-sativene sesquiterpeno id	Helminthosporic acid, Cochliobolin F, Helminthosporal acid, and Drechslerine B	*Cochliobolus sativus*	Phytotoxicity against phytopathogens	Macías-Rubalcava and Garrido-Santos (2022)
Non-volatile compounds	Hormones	Gibberellins, Indole acetic acid	*Penicillium funiculosum* LHL06*, Penicillium* spp. CBRF65	Bioremediation, Plant growth promotion	Badenoch-Jones et al. (1984)
	Enzymes	Glutathione S-transferase	*Penicillium funiculosum* LHL06	Bioremediation	Badenoch-Jones et al. (1984)
	β-resorcylic acid derivatives	de-O-methyllasiodiplodin,14-hydroxy-de-O-methyllasiodiplodin, ethyl 2,4-dihydroxy 6-(8-hydroxyheptyl) benzoate	*Lasiodiplodia theobromae* strain GC-22	Phytotoxicity against phytopathogens	Macías-Rubalcava and Garrido-Santos (2022)
	Other Natural organic compounds	Isocoumarins	*Phomopsis prunorum*	Phytotoxicity against phytopathogens	
		Chromenone and derivatives	*Daldinia eschscholtzii*	Phytotoxicity against phytopathogens	
		Rhizoperemophilanes,1α- hydroxyhydroisofukinon, PR-toxin dimethyl acetal	*Rhizopycnis vagum*	Phytotoxicity against phytopathogens	
		Fumigaclavine A, Fumigaclavine B, and Fumigaclavine C	*Aspergillus oryzae*	Resistance against phytopathogens	
		Mycotoxin deoxynivalenol	*Enterobacter* spp.	Resistance against phytopathogens	Etesami et al. (2021)
		Glomalin	*Glomus* spp.	Increased nutrient uptake,	Etesami et al. (2021)
		Siderophores	*Aspergillus* spp., *Pseudomonas* spp., *Paracoccidioides* spp. *Saccharomyces cerevisiae, Gliocladium virens, Rhodothamus chamaecistus*	Increased iron acquisition from soil and more availability of iron, resistance against phytopathogens	Silva et al. (2020)
		Harzianum A	*Trichoderma harzianum*	Fungicides, resistance against phytopathogens and biostimulants	Khan et al. (2023)

#### 2.3.2 Role of volatile and non-volatile compounds in post-harvest disease management

The role of endophytic bioactive compounds in plant growth, defense, and sustainable agriculture has been well documented in literature (Santra and Banerjee, 2023). The post-harvest loss of crop plants due to pathogens or physiological conditions is high, especially in the case of fruits and vegetables. With the growing resistance of phytopathogens to traditional anti-pathogenic agents like fungicides, pesticides or insecticides etc., and the effects of such substances have shifted the focus towards more sustainable options. The use of alternative approaches such as low temperatures, irradiation, essential oils, salt, antagonistic microorganisms etc. for post-harvest protection of crop plants has been documented (Youssef et al., 2022) The use of endophytic fungi as a biocontrol agent for both pre- and post-harvest protection of crop plants has emerged as a viable alternative for various chemical compounds like pesticides, insecticides, herbicides etc. (Kumar et al., 2021). Fungal endophytes such as members of genera *Muscodor*, *Xylaria*, *Trichoderma*, *Fusarium* etc. are known to produce a large number of volatile compounds with the potential of post-harvest crop protection (Macías-Rubalcava and Garrido-Santos, 2022). The volatile compounds produced by the endophytic fungi can be applied as fumigation agents, for creating controlled atmospheric storage or as inhibitors of hormones that promotes the ripening of the stored crop. Volatile compounds such as esters, alcohol hydrocarbons, lipids, ketones acids, etc. are more suitable, effective, and ecologically sustainable for the management of post-harvest pathogens due to their long-distance antagonistic action scale, resulting in direct penetration at spatial scales, e.g., volatiles of *M. albus* (also known as Mycofumigation,), volatiles of *Oxyporus latemarginatus*, volatiles of *Nodulisporium* which inhibits 12 different pathogens (Naik, 2018) Similarly, many non-volatile compounds produced by endophytic fungi are exploited for their potential of controlling the post-harvest pathogens. Compounds like ergot alkaloids produced by *Clavicipitaceae* have antimicrobial and pesticidal properties that are effective against a number of post-harvest pathogens (Florea et al., 2017). Resorcyclic acid lactones produced by *Penicillium* spp., Zopfiellin produced by *Zopfiella* spp. Chaetoglobosins are produced by *Chaetomium* spp. are the examples of non-volatile compounds that possess antimicrobial properties. Other compounds like aflavinines produced by *Aspergillus* spp., Indole-Diterpenes produced by *Clavicipitaceae*, Harzianic acid produced by *Trichoderma* have anti-fungal properties, Peramine produced by *Epichloë*, Tetramic Acid Derivatives produced by different genus of endophytic fungi posses’ properties such as insecticidal, fungicidal and antimicrobials thus, promoting crop protection post-harvest by providing defense against the phytopathogenic (Song et al., 2021). The role of volatile and non-volatile compound in post-harvest plant protection is paramount. Use of these compounds not only help in post-harvest disease management of the crops but also offer a sustainable agriculture practice. These compounds offer multifaceted solutions that address the complex challenge of controlling pathogens and extending the shelf life of harvested crops.

### 2.4 Phosphate solubilization

For the growth and development of plants, phosphorus is a crucial nutrient. However, in most soils, phosphorus is present in insoluble forms like phosphate rocks or mineral complexes. This renders it less available to plants, limiting their growth and overall health (Johan et al., 2021). Endophytic fungi have evolved the ability to solubilize phosphorus from these insoluble forms into soluble forms that the plant can readily absorb. This is achieved through the secretion of organic acids, which are powerful chelators capable of breaking down complex phosphate compounds. One of the primary organic acids produced by endophytic fungi is gluconic acid. This acid is particularly effective in dissolving phosphorus compounds in the soil. When the endophytes release gluconic acid into the plant’s rhizosphere, it reacts with the insoluble phosphorus, converting it into soluble phosphate ions. These soluble phosphate ions become more available for uptake by the plant’s root system (Etesami et al., 2021). Phosphate solubilization by endophytic fungi is a crucial process that aids in protecting plants from pathogens. By facilitating phosphorus uptake, endophytic fungi contribute to improved plant health and vigor (Baron and Rigobelo, 2022) and healthy plants are better equipped to defend themselves against pathogens. When a plant is well-nourished with an ample supply of phosphorus, it can allocate more energy and resources toward its defense mechanisms. The mechanisms employed by endophytic fungi for phosphate solubilization can be categorized into acidification, enzyme activity, and ion exchange (Sharma et al., 2013). Phosphate solubilization by acidification involves the secretion of organic acids like citric acid, oxalic acid, malic acid, gluconic acid etc. into the rhizosphere. These organic acids act as chelating agents for the binding of metal ions present in the soil to facilitate the release of bound phosphate, thus making them available for plant uptake. Fungal strains such as *Trichoderma viride*, *Trichoderma harzianum*, *Trichoderma vixens*, and *Trichoderma longibrachiatum* have been reported for phosphate solubilization by acidification (Elhaissoufi et al., 2022). Many endophytic fungi such as *Penicillium* and *Aspergillus* have been shown to produce various enzymes like phosphatases and phytases which are capable of hydrolyzing the organic phosphate compounds present in soil and converting insoluble phosphate into soluble inorganic phosphate (Chaudhary et al., 2022). Other than these two mechanisms, endophytic fungi can also exchange metal cations present in the rhizosphere with phosphates, thus effectively releasing soluble phosphates into the surrounding soil (El Hassan, 2017). Endophytic fungi provide an additional source of phosphorus to the plants by solubilizing phosphate, thus enhancing their nutrient uptake and promoting overall growth and health. This increased nutrient availability makes the plants more resistant to pathogenic attacks in several ways such, as enhanced plant growth, and induced systemic resistance like phosphate-solubilizing endophytic fungi can trigger systemic resistance in plants. They can stimulate the plant’s defense mechanisms and produce various secondary metabolites that act as natural biopesticides, thereby protecting the plant from pathogenic attacks. Overall, phosphate solubilization by endophytic fungi is a multifaceted mechanism that contributes significantly to plant protection against pathogens. These beneficial fungi play a vital role in promoting plant health and crop productivity while reducing the need for chemical fertilizers and pesticides, making them essential components of sustainable agriculture practices.

### 2.5 Siderophore production

For all eukaryotes and almost all prokaryotes, iron is a crucial nutrient, since it is necessary for metabolic function. Availability of iron in two different oxidation states, i.e., ferric (Fe^3+^) and ferrous (Fe^2+^), helps in serving iron both as a cofactor and a catalyst in basic metabolic processes. Despite being one of the metals with the highest abundance on Earth, iron has a low bioavailability due to the fact that iron forms a highly insoluble compound ferric hydroxides in the presence of oxygen (Expert, 1999). Overall, a sufficient supply of iron is necessary for survival. Various species have created controlled systems to maintain homeostasis between sufficient uptake of iron and preventing iron toxicity to avoid cell damage. In plants, animals, and bacteria, iron with high-affinity forms complex with glycoproteins such as lactoferrin or transferrin. Iron is stored intracellularly in ferritin (Arosio and Levi, 2002). Before recent times, little was known about the role of iron in interactions between fungi and their hosts as well as the general regulatory mechanisms that control iron homeostasis. Reductive iron assimilation and Siderophore-mediated iron uptake are the two major systems used by fungi for iron uptake (Oide et al., 2006). Microbes produce siderophores, which are low molecular weight, essentially ferric-specific ligands, as scavenging agents to counteract low iron stress (Sid = Iron, Phores = Bearers; Korat et al., 2001). Except for Lactobacilli, *Candida albicans, Cryptococcus neoformans*, *Saccharomyces cerevisiae* all other aerobic and facultative anaerobic microorganisms are known to produce siderophores, which function as iron chelates (Loper and Buyer, 1991). Siderophores are thought to be the result of Fe^2+^ being simultaneously oxidized to Fe^3+^ and precipitated as ferric hydroxide as an evolutionary reaction to the presence of O2 in the atmosphere (Winkelmann and Drechsel, 2008). Variety of siderophores produced by fungi have been classified mainly into five major classes (a) fusigens, (b) coprogens, (c) ferrichromes, (d) rhodotorulic acid, and (e) rhizoferrin (Chincholkar et al., 2000). Some of the fungal siderophores are enlisted in Table 3.

**Table 3:** List of reported siderophores produced by various endophytic fungi.

S. no.	Siderophore	Reported from	References
1.	Alterobactin	*Alteromonas luteoviolaces*	Sayyed et al. (2013)
2.	Asperchrome A, B & C	*Aspergillus orhraceus*	Sayyed et al. (2013)
3.	Coprogen	*Curvularia lunata*	Yuan et al. (2001)
4.	Canadaphore	*Helmenthosporium carbonum*	Sayyed et al. (2013)
5.	Ferrichrome A	*Ustilago sphaerogena*	Yuan et al. (2001)
6.	Ferrichrome	*Ustilago maydis*	Yuan et al. (2001)
7.	Ferricrocin & Hyperferricrocin	*Aspergillus fumigatus*	Schrettl et al. (2007)
8.	Rhizoferrin	*Rhizopus microsporus*	Yuan et al. (2001)
9.	Rhodotorulic acid	*Rhodotorula piliminae*	Johnson (2008)
10.	Triacetyl- fusarinine C	*Aspergillus fumigatus*	Schrettl et al. (2007)

The extraordinarily high affinity of siderophores for ferric ions is the most notable characteristic of Siderophores (Askwith et al., 1996). When competing for nutrients in the soil, the ability to exploit siderophores produced by different microbes is a significant selection advantage. The most significant biotechnological importance of siderophores is in the plant’s rhizosphere, where they nourish the plant with iron, act as a first line of defense against parasites that invade the roots, and aid in the removal of hazardous metals from contaminated soil (Matzanke et al., 1987). Studying the opportunistic human pathogen *Aspergillus fumigatus* led to the initial discovery of the function of fungal siderophores in illness (Sindhu et al., 1997). *In vitro* removal of iron from transferrin by siderophores was linked to the survival of pathogen in human serum (Hissen et al., 2004). Another example of role of fungal siderophores can be seen in *Candida albicans*, a non-siderophore producing yeast. *Candida albicans* requires Ferrichrome-type siderophores along with Arn1p/Sit1p, a siderophore transporter, for invasion and penetration of its host epithelia (Heymann et al., 2002). The discovery that the metabolic byproduct of the biosynthetic pathway involving the NPS6 gene, encoding a non-ribosomal peptide synthase from *Cochliobolus heterostrophus*, a phytopathogenic fungus, recognized the role of Siderophores in fungal virulence towards plants (Oide et al., 2007). Many studies on NPS6 gene demonstrated its role in virulence to maize against H_2_O_2_ hypersensitivity and to be broadly conserved among the filamentous ascomycetes (Lee et al., 2005). Beyond virulence, fungal siderophores also serve to maintain the mutualistic symbiotic relationships between grass and endophytes. The grass symbiont *Epichloe festucae* uses the NRPS gene sidN to make a new extracellular siderophore that looks like fusarinine-type siderophores. These helpful fungi are never free-living; instead, they are restricted to the intercellular spaces (apoplast) of leaf sheaths and blades, where they do not spread disease. On the other hand, Mycorrhizal fungi frequently create advantageous symbiosis with the roots of terrestrial plant communities, which benefit plant nutrition, including the uptake of micronutrients (Johnson, 2008). Also, these fungi have been found to produce hydroxamate siderophores, and it is believed that siderophore-mediated iron uptake is crucial for the acquisition of iron by the host plant (Haselwandter et al., 2006). Thus, the production of siderophores is a potent strategy employed by various fungal endophytes to grab iron from the environment. Both extracellular and intracellular siderophores play an essential role in numerous fungal-host interactions.

### 2.6 1-Aminocyclopropane-1-carboxylate utilization

Plant hormones take an active role in symbiotic, defensive, and developmental processes. Ethylene (ET), a gaseous plant hormone that is easily absorbed by plant tissues and has effects even in extremely low quantities, is at the center of these activities (Van de Poel et al., 2015). Some studies have explored the impact of ethylene on the cell division and the finding indicates that ethylene can exert contrasting effects on the cell cycle depending on the specific tissue as well as internal and external stimuli. During the development of the apical hook, ethylene drives cell division in the subepidermal layers, most likely working in conjunction with auxins (Van de Poel et al., 2015). In addition to controlling several aspects of plant growth and development, Ethylene also engages in microbial defense and symbiotic programs which affect overall microbial assembly (Desbrosses and Stougaard, 2011). Moreover, ACC (1-Aminocyclopropane-1-carboxylate) a major precursor of ethylene is a non-proteinogenic α-amino acid that has been reported to play an important role in regulating numerous plant developmental and defense responses. The production of ethylene precursor is mainly regulated at all the major levels (transcription, post-transcription, translation, and post-translation; Lee et al., 2017). The molecule is produced from S’ adenosyl methionine (SAM) in a reaction catalyzed by an enzyme ACC-synthase, releasing MTA (5-methylthioadenosine). Through a series of biochemical reactions, the released MTA is reconverted to methionine to renew the stack of available methionine (Bennett, 2003). Being localized in the cytosol, ACS is one of the members of the Pyridoxal phosphate (PLP) dependent enzymes that uses vitamin B6 as a co-factor for its enzymatic activity (Boller et al., 1979). MACC (malonyl-ACC; Amrhein et al., 1981), GACC (γ-glutamyl-ACC; Martin et al., 1995) and JA-ACC (Jasmonyl-ACC; Staswick and Tiryaki, 2004) are three different conjugates of ACC namely suggesting a complex overall biochemical regulation of ACC pool with eventual effects on the production of ethylene and other developmental and physiological processes. Apart from these conjugates produced in different mechanisms, another distinctive way to metabolize ACC (1-Aminocyclopropane-1-carboxylate) is the deamination of ACC. Initially, ACC deaminase was first reported in bacteria. Many reports suggested that some plant growth-promoting bacteria have the potential to process the plant-based ACC using the ACC deaminase enzyme into ammonia and α-ketoglutarate (Honma and Smmomura, 1978). Being multimeric with an approximate subunit molecular mass of 35-42 kDa (Ullah et al., 2019), ACC deaminase is a sulfhydryl enzyme that requires pyridoxal 5’-phosphate (PLP) for its enzymatic activity (Kushwaha et al., 2020). The cleavage of ACC to ammonia and α-ketoglutarate catalyzed by the enzyme ACC deaminase was discovered in 1978 (Ullah et al., 2019). Following X-ray crystallographic studies, the enzyme ACC deaminase folds into two domains, each of which has an open twisted α/β structure resembling the β-subunit of the tryptophan synthase (Husen et al., 2008). This PLP-dependent enzyme has a low affinity for ACC with a 1.5-15 mM reported Km value (Hontzeas et al., 2004). Many studies have shown that the root exudates contain an explicit amount of ACC that might attract ACC deaminase harboring microorganisms and set up Rhizospheric interaction (Glick et al., 2007). Interaction with the root environment is a must to access the plant-based ACC by the plant growth-promoting microbes containing ACC deaminase enzyme (Payment et al., 2011). Apart from bacterial species, fungi are also exploited for their ACC deaminase activity. Even though the functional principles of the bacterial and fungal ACC deaminase are substantially the same, their structures differ due to sequence variations. Comparison of AcdS gene sequences of various fungal and bacterial strains have shown that approximately 70-90% sequence similarity was shown by the fungal strains with that of bacterial AcdS gene rather than with the fungal gene sequence. *Schizosaccharomyces pombe* 972 h, *Clavispora lusitaniae* ATCC 42720, *Cyberlindnera saturnus* are some of the major examples of fungal strains with higher sequence similarity with bacterial AcdS rather than fungal. It has been hypothesized that horizontal gene transfer is responsible for the transfer of genes from Proteobacteria to the above-mentioned fungal strains (Nascimento et al., 2014) Furthermore, the isolated fungal ACC Deaminase genes similar to Proteobacteria mainly belong to classes Ascomycota and Basidiomycota (Gravel et al., 2007).

### 2.7 Competition with pathogens

In the rhizosphere and phyllosphere of plants, competitive exclusion, a frequent phenomenon, controls the population of a wide range of species (Sikora et al., 2007). In some circumstances, the biological control of root diseases and nematodes is also a result of competition for nutrients and space. Therefore, when several inoculants are required for efficient pest management, the “first-come, first-serve” aspect of colonization and the idea of survival of the fittest are the major factors that could affect the efficacy of biological control (Backman and Sikora, 2008). Because nutrients in soils are typically in scarce supply and difficult to acquire, competition for resources such as oxygen, nutrients, etc. is the active demand between soil-inhabiting microorganisms including pathogens and non-pathogens, and is a key mechanism for the management of diseases that are carried by soil (Stoytcheva, 2011). Due to competition for scarce nutrients and the fact that starvation is a significant and frequent cause of microbiological death, fungal phytopathogens may be biologically controlled (Eisendle et al., 2004). If the requirements both in terms of nutrients, oxygen, space of endophytic fungi, and the disease-causing pathogens are similar, then only competition will occur among them. Species of *Fusarium* and *Pythium*, soil-borne pathogens that infect plants through mycelial contact are more prone to competition than those that invade through infection threads (Van Dijk and Nelson, 2000).

#### 2.7.1 Competition for Iron

Iron is the most abundant element on Earth and an essential nutrient for microorganisms. Though it is the fourth largest mineral found abundantly on our planet it is not readily available to microorganisms. As it gets oxidized easily, it is mainly available in the form of ferric ions that cannot be directly utilized due to its extremely low solubility (Neilands et al., 1987). This in turn leads to competition among microorganisms. One of the biological controls for both bacterial and fungal phytopathogens is the fight for iron nutrition (McMorran et al., 2001). Several studies have indicated that the concentration of iron in the soil is notably lower than the necessary level for microbial growth. Siderophores, known as iron chelators, are low molecular weight molecules that bind to ferric ions dueto their high affinity for iron with Kd values ranging from 10^-20^ to 10^-50^ (Castignetti and Smarrelli, 1986). Thus, siderophore-producing microbial strains have a selective advantage over the non-producing strains, including disease-causing microorganisms. This results in the inhibition of the growth of pathogens in their immediate proximity due to iron limitation (O’Sullivan and O’Gara, 1992). It has been found that the rhizosphere biological control agents protect plants from pathogens generally by colonization resulting in the consumption of already available substrates in their surroundings. This makes disease-causing microorganisms or pathogens difficult to grow. Siderophores are produced by a wide range of Plant Growth Promoting Rhizobacteria such as *Serratia* (Berg et al., 2005), *Bacillus* (Yu et al., 2011), *Pseudomonas* (Elad and Baker, 1985) and many more playing major roles in suppressing soil-borne disease-causing pathogens. Based on the ligands, siderophores are mainly classified as carboxylates, phenolates (Catecholates), or hydroxamates (Winkelmann and Drechsel, 2008). Enterobactin, Siderochelin A, Ferrocins, Pyoverdines, Acinetobactin, Cepabactin are among the major examples of bacterial siderophores whereas Ferrichromes, Coprogens, Rhodotorulic Acid, Fusigens and Rhizoferrins are the typical examples of siderophores produces by fungal strains (Winkelmann and Drechsel, 2008). Among these Siderophores, Catecholates are exclusively produced by bacteria but hydroxamate siderophores are produced both by bacteria and fungi (Cox et al., 1981). Many studies revealed that the fungal phytopathogens have a lower affinity for iron as compared to Plant Growth Promoting Rhizobacteria (PGPRs) and thus overpower the fungal phytopathogens for iron (Schippers et al., 2003). In such a case, *Pythium and Fusarium* species are more prone to competition for iron as they cause infection through mycelial contact, from other microbes associated with soil and plants than those species that germinate directly on plant surfaces (Pal and Gardener, 2006). By analyzing the siderophore mutants, the role of siderophores in biocontrol either alone or in combination with other metabolites such as antibiotics has also been studied (Buysens et al., 1994). Many factors are responsible for affecting the overall capability of siderophores such as properties of soil, type of plant, type of siderophore, type of microbial strain, and type of disease-causing pathogens (Glick and Bashan, 1997). The plant diseases caused by various pathogens such as *Fusarium oxysporum*, *Rhizoctonia solani*, *Pseudomonas* sp., *Alternaria* sp. etc. are suppressed by siderophores in numerous reports (Sahu and Sindhu, 2011). This overall suggests that to overpower the soil-borne disease-causing pathogens, competition for iron and other mineral ions is one of the major mechanisms, however, siderophores only may not be able to suppress the disease in any case.

#### 2.7.2 Competition for niches

In the biocontrol mechanism of fungal endophytes, competition for niches is how different fungal endophytes fight for space and resources inside a plant host in order to become dominant and use their biocontrol effects against pathogenic fungi. It has long been understood that niche complementarities may play a significant role in a species’ ability to coexist (Backman and Sikora, 2008). The endosphere and the rhizosphere are the complex ecosystems that play a significant role in supporting the overall growth and development of the plants. A wide variety of microorganisms, including fungi, are heavily concentrated in the rhizosphere, the soil environment around plant roots, and the endosphere, which consists of the internal tissues of plants. The two fungal groups—endophytes and plant pathogenic fungi—represent opposing forces in this ecosystem, competing with one another for the little resources and ecological niches available (Oszust et al., 2020). In the endosphere and rhizosphere, competition for resources and space can be fierce, and it is influenced by a number of important variables. First, competition may arise from direct hostility between the two fungal species, which frequently takes the form of mycoparasitism, the direct use of resources, or the synthesis of antimicrobial substances. For instance, the well-known mycoparasitic properties of the endophyte *Trichoderma* allow it to actively attack and parasitize pathogenic fungi such as *Rhizoctonia solani,* thereby lessening their detrimental effects on plant health (Howell, 2003). It has also been discovered that fungi in the genus *Trichoderma* have an ecological niche that is most similar to that of *Colletotrichum* spp., hence excluding the latter phytopathogenic species (Oszust et al., 2020). *Trichoderma* spp. is assumed to be tough competitors that drive out slower-growing diseases since they are discovered as being particularly quick colonists (Tyśkiewicz et al., 2022). Based on an *in vitro* experiment, Oszust et al. (2020) discovered that *Trichoderma* spp. nutritionally outcompeted *Botrytis* sp., *Verticillium* spp., and *Phytophthora* spp. According to Morandi et al., the non-pathogenic endophyte *Clonostachys rosea* inhibited the growth and sporulation capability of *B. cinerea* to regulate it (Morandi et al., 2000). Second, in moderating the conflict between plant diseases and endophytes, plant host selection is essential. Certain plants have the ability to actively attract or encourage the growth of particular endophytes, which benefit them and restrict harmful fungus. In order to give endophytes a competitive edge, this procedure may involve the release of root exudates that specifically favor advantageous fungus. Moreover, the outcome of the rivalry between the two fungal groups can also be determined by their spatial distribution. The vascular system and intercellular gaps are two examples of the particular niches that endophytic fungi frequently occupy within plant tissues (Faeth, 2002). Due to the possibility that they have distinct preferred areas for colonization, this spatial separation can reduce direct competition with plant pathogenic fungus. On the other hand, direct interactions between endophytes and pathogenic fungi are more likely to occur in the rhizosphere, which is the interface between plant roots and soil. Researchers and agricultural scientists are interested in understanding these interactions because they can impact the efficacy of using biocontrol endophytes as a sustainable alternative. By studying the competition for niches among fungal endophytes and their interactions with pathogens and the host plant, they can develop strategies with pathogens and the host plant to enhance the biocontrol potential of specific endophytes, and improve plant disease management practices.

## 3 Indirect mechanisms of plant protection

To survive in harsh environments like famine, salt stress, and cold, plants use a variety of strategies. Some of the biochemical and morphological changes that can be seen right away are the formation of phytoalexins, the death of cells, and the hypersensitive response. Kiraly et al., found that long-term evolution can lead to both non-specific (generic) and specific (pathogen-specific) resistance (Kiraly et al., 2007). Plants with non-specific resistance can protect themselves from a broad range of diseases, but plants with particular resistance can avoid getting sick from just one or a few infections. Endophytes produce secondary metabolites and have improved resistance, which strengthens the plant’s defense system.

### 3.1 Induced plant resistance

Several research have examined how plants respond to disease and parasite invasions for more than 20 years, utilizing several categories. Systemic acquired resistance (SAR) and induced systemic resistance (ISR) are the two types of resistance that researchers are most interested in. ISR is regulated by ethylene or jasmonic acid, which is produced by some non-pathogenic rhizobacteria and not connected to the accumulation of pathogenesis-related (PR) proteins. Salicylic acid mediates SAR, which is a result of pathogen infections and is linked to the production of PR proteins (Tripathi et al., 2008). Invading cells are directly lysed by these PR proteins’ many enzymes, like chitinases and 1, 3-glucanases, which also strengthen cell wall borders and increase resistance to cell death and infection (Gao et al., 2010). ISR generated by endophytes has also been associated with increased expression of genes involved in disease. The ISR process does not directly kill the virus or restrict it. Instead, it strengthens the plants’ natural physical or chemical barriers (Van Loon et al., 1998). ISR and SAR frequently have an antagonistic action that controls the signaling at the cellular level. When SA and JA have an antagonistic effect on biotrophic or necro pathogens, and vice versa, there is upstream and downstream signaling between them (Syed Ab Rahman et al., 2018). For instance, *Blumeria graminis* f. spp. *Tritici*, which causes powdery mildew in wheat, is controlled by *Bacillus subtilis* by inducing disease resistance via the SA-dependent signaling pathway (Xia et al., 2022). The non-expression of the pathogenesis-related genes 1 (NPR1 and NPR3/4), which eventually trigger an antagonistic response via SAR through priming and reveal resistance against secondary infections, is crucially regulated by the pathogenesis-related gene 1 (PR1; Ding et al., 2009). By preventing *Botrytis cinerea’s* spore germination and mycelium growth through the ISR method of resistance, *Burkholderia* species (BE17 and BE24) shield grapevine from the grey mold disease (Lahlali et al., 2022). *Trichoderma* spp. AA2 and *Pseudomonas fluorescens* PFS are the most powerful antagonists of *Ralstonia* spp., which causes bacterial wilt in tomatoes by generating ISR in the plant.

### 3.2 Stimulation of plant secondary metabolites

Secondary metabolites are bioactive substances that have an important function in ecological interactions, competition, and defensive signaling (Liu et al., 2018). The production of secondary metabolites via a metabolic exchange, which exhibits a complicated regulatory response, is necessary for the formation of microbial contact. These interactions may be competitive, parasitic, mutualistic, hostile, or mutualistic. The most recent advances in imaging mass spectrometry (IMS) technology have been employed to investigate the diverse roles played by mold metabolites in microbial interactions. The growth of phytopathogens is regulated by the antibacterial and antifungal activities of secondary metabolites. While under biotic stress, plants can create secondary metabolites on their own or in collaboration with other endophytes to manage stress and mount defenses (Ludwig-Müller, 2015). To safeguard plants and enhance crop quality, endophytic secondary metabolites are employed as a biocontrol agent. Whereas microorganisms create homogeneous, high-quality metabolites with the highest efficacy in terms of their biocontrol potential, plants produce bioactive molecules that are inadequate and variable in quality (Lugtenberg et al., 2016). An example of plant secondary metabolite stimulation by endophytic fungi is the production of loline (an alkaloid) in the leaves of fescue grasses by endophytic fungus *Neotyphodium* spp. and *Epichloë* spp., which protects the leaves from herbivores. The interaction between endophytic fungus *Neotyphodium coenophialum* and its host plant is another example of plant secondary metabolite stimulation. *Neotyphodium coenophialum* protects its host plants from aphids the key carriers of viruses, e.g., protection of *Festuca arundinacea* against *Rhopalosiphum padi* also inhibit the spread of viruses (Alam et al., 2021).

### 3.3 Hyperparasites and predation

Hyperparasites are another means by which endophytes protect their host ecologically. In this approach, recognized pathogens or their zoospores are immediately attacked by endophytes (Tripathi et al., 2008) By twisting and piercing the hyphae of the pathogens and creating lyase, which dissolves the pathogen’s cell wall, endophytic fungi trap the pathogens. This sort of interaction among fungi is frequently seen. Hyper parasitism in bacteria has only occasionally been documented. A predatory bacterium called *Bdellovibrio bacteriovorus* has the peculiar ability to exploit the cytoplasm of other Gram-negative bacteria as food (McNeely et al., 2017). Over 30 fungal species, including *Cladosporium uredinicola* against *Puccinia violae* and *Alternaria alternata* against *Puccinia striiformis* f. spp. *tritici*, reported by Zheng et al. to exhibit hyper parasitism against rust pathogens (Jia et al., 2016). The reduction of plant pathogens through microbial predation is another technique. The majority of endophytes show their predatory traits in nutrient-poor environments. For example, fungal endophytes isolated from *Taxillus chinensis* produce cell wall-degrading enzymes that promote the dissolution and relaxation of the cell wall between the host and *Taxillus chinensis* (Wu et al., 2021).

### 3.4 Fungal endophytes as biocontrol agents

Endophytes are described as microorganisms (bacteria or fungal) that are found living in tissue, plant organs, and seeds of almost all vascular plants (Caruso et al., 2022). The interaction between the endophyte and the host plant during their association has proven to be beneficial rather than harmful for both interacting organisms. These benefits are always based on the interaction between endophytes and the host plants (Kamana et al., 2016). Many novel bioactive compounds like antibiotics, antimycotics, antineoplastics, etc. are endophytic products (Shukla et al., 2014). The agro-industry relies heavily on the use of agrochemicals for controlling phytopathogens. This excessive use of chemicals in the agricultural industry has resulted in the development of resistant phytopathogens. The endophytes play an important in maintaining the health of the host plant (Wu et al., 2021). These endophytes can be exploited as a biocontrol agent in the agro-industry. Biocontrol agents (BCA) are described as living organisms or their products that can fight against plant diseases or pests via direct antagonistic action (Xia et al., 2022). Many endophytes are being exploited as BCA as they are effective in controlling plant diseases and can help attain sustainable agriculture. The endophytic fungi that act as biocontrol agents against different phytopathogens are summarized in Table 4.

**Table 4:** Summarized list of endophytic fungi that act as biocontrol agents against various phytopathogens.

S. no.	Endophytic fungi	Phytopathogen activity	References
1.	*Aspergillus niger*	*Colletotrichum acutatum*	Landum et al. (2016)
2.	*Anthrinium* sp.	*Colletotrichum acutatum*	Landum et al. (2016)
3.	*Amphirosellinia nigrospora*	*Ralstonia solanacearum, Magnaporthe oryzae*	Nguyen et al. (2019)
4.	*Aureobasidium pullulans*	*Fusarium oxysporum f.* spp. *Herbemontis*	Adeleke et al. (2022)
5.	*Aporospora terricola,*	*Fusarium oxysporum f.* spp. *Herbemontis*	Adeleke et al. (2022)
6.	*Aspergillus flavus*	*Mycotoxigenic Aspergillus*	Camiletti et al. (2018)
7.	*Bjerkandera adusta*	*Fusarium oxysporum f.* spp. *Herbemontis, Colletotrichum gloeosprioides*	Adeleke et al. (2022)
8.	*Beauveria bassiana*	*Parasitic Nematodes, Botrytis cinerea, Alternaria alternate, Macrosiphum euphorbiae, Tribolium confusum*	Gautam and Avasthi (2019)
9.	*Colletotrichum boninense*	*Fusarium oxysporum f.* spp. *Herbemontis,*	Adeleke et al. (2022)
10.	*Clonostachys rosea*	*Fusarium culmorum, Botrytis cinerea, Nematodes, Sclerotinia sclerotiorum*	Dubey et al. (2020)
11.	*Conidia prior*	*Puccinia triticina, P. hordei*	Wilson et al. (2020)
12.	*Colletotrichum gloeosporioides*	*Fusarium oxysporum, Fusarium culmorum, Sphaceloma* sp., *Sordariomycetes, Glomerella* spp.	Adeleke et al. (2022)
13.	*Diaporthe citri*	*Fusarium solani, Glomerella* spp.*, Moniliophthora perniciosa*	Santos et al. (2019)
14.	*Epicoccum nigrum*	*Colletotrichum acutatum, Gibberella, Haematonectria, Fusarium graminearum*	Ogórek et al. (2020)
15.	*Flavodon flavus*	*Alternaria* spp.*, Glomerella* sp., *Fusarium culmorum,*	Adeleke et al. (2022)
16.	*Fusarium oxysporum*	*Pythium ultimum, Verticillium dahlia*	Fadiji and Babalola (2020)
17.	*Lecanicillium lecanii*	*Thrips, Whitefly, Aphid, Macrosiphum euphorbiae (Thomas), Scales, Mealybugs*	Reddy (2020)
18.	*Metarhizium anisopliae*	Parasitic Nematodes, *Protaetia brevitarsis seulensis*	Gautam and Avasthi (2019)
19.	*Purpureocillium lilacinum*	*Parasitic nematodes and insects*	Wang et al. (2016)
20.	*Paenibacillus polymyxa*	*Aspergillus aculeatus*	Liu et al. (2018)
21.	*Piriformospora indica*	*Golovinomyces orontii*	Grabka et al. (2022)
22.	*Rosellinia bunodes*	*Bacillus subtilis, Staphylococcus aureus*	Becker and Stadler (2020)
23.	*Serendipita herbamans*	*Fusarium oxysporum*	Sefloo et al. (2021)
25.	*Trichoderma viride*	*Penicillium digitatum, Phytophthora nicotianae, Rhizoctoniasolani*	Fontana et al. (2021)
26.	*Xylaria feejeensis*	*Alternaria tomatophila*	Brooks et al. (2022)

### 3.5 Protection against leaf-cutting ants

Endophytic fungi play a pivotal role in safeguarding plants from the destructive foraging of leaf cutting ants. These mutualistic fungal endophytes, which commonly inhabit plant tissues, establish intricate associations with their host plants. One of their primary mechanisms of protection lies in the production of secondary metabolites, such as alkaloids and mycotoxins, which serve as potent deterrents against herbivores, including leaf-cutting ants (White and Torres, 2010). By secreting these chemical compounds, endophytic fungi effectively shield their host plants, rendering them unpalatable or toxic to the ants. This protective alliance not only benefits the plants by reducing herbivore-induced damage but also highlights the fascinating interplay between plants and their endophytic partners in the intricate web of ecological relationships.

#### 3.5.1 Hypocreales endophytes in coffee plants

Fungal endophytes belonging to the order Hypocreales have been discovered in coffee plants, producing mycotoxins that deter leaf cutting ants. The presence of these endophytes in the coffee plants was demonstrated in a study conducted by Mejia et al. in the year 2008 (Mejía et al., 2008).

#### 3.5.2 Trichoderma species

Certain Trichoderma species are endophytic fungi that have been shown to protect plants from various pests, including leaf-cutting ants. These endophytes can produce antifungal and insecticidal compounds that deter herbivores. Studies like Lo et al. (2015) have highlighted their potential in biocontrol (Yao et al., 2023).

#### 3.5.3 Clavicipitaceous endophytes in grasses

Clavicipitaceous fungal endophytes, such as *Neotyphodium* and *Epichloe* species are known to form mutualistic associations with grasses. They can produce alkaloids, like ergot alkaloids, that deter herbivores including leaf-cutting ants (Saikkonen et al., 2004).

These examples highlight the diverse array of fungal endophytes and their crucial role in protecting plants from leaf-cutting ants through various mechanisms, including the production of chemical compounds and mutualistic associations.

## 4 Conclusive remarks

In light of the aforementioned explanation, endophytic fungi mostly use direct and indirect approaches to combat disease-causing pathogens. The management and treatment of plant diseases are extremely important for sustainable agriculture. In order to prevent adverse impacts on the environment and human health, national rules are becoming harsher when it comes to regulating and authorizing new pesticides. The demand for organic and safe foods is growing daily. It is therefore vital to look for novel control strategies that ensure food safety and lessen these adverse consequences. To keep the harm caused by herbivores or pathogens at manageable levels for farmers, a worldwide strategy that considers all of the aforementioned issues must be devised. In this regard, with emerging applications in agriculture, endophytes present a very intriguing subject of study. Undoubtedly, a variety of endophytic fungi can be exploited for the treatment of various plant diseases and as a substitute for available biocontrol agents against phytopathogens. These fungal endophytes are also a good source of beneficial bioactive substances for plants. However, not much attention has been paid to how important it is for biotechnology that endophytic fungi make beneficial metabolites that help control plant diseases. In recent times, biologically active compound producing and plant growth promoting endophytes have drawn more interest from researchers since they are potential sources of novel drugs and eco-friendly plant protectors. Among all identified possible biocontrol agents, members of the genus *Trichoderma* have received extensive investigation; however, there are many other comparably potent endophytic fungal families that have not been adequately investigated and explored. These biocontrol organisms have a significant impact on agriculture and the overall environment. Molecular identification of microbes which are being exploited as biocontrol agents and biological characterization of bioactive chemicals produced by them is essential for understanding their antagonistic mechanisms.

## 5 Future prospects

The future prospects of biocontrol mechanisms involving fungal endophytes in sustainable agriculture appear exceptionally promising. As the world grapples with the growing need for environment friendly and resilient farming practices, fungal endophytes stand out as a valuable ally. These fungi possess the ability to bolster crop health and protect against pests and pathogens, reducing the reliance on chemical pesticides. As the understanding of the intricate interactions between fungal endophytes and plants deepens, can expect the development of specialized, genetically optimized strains that offer even greater efficacy. This could lead to substantial reductions in agriculture’s environmental footprint while simultaneously increasing crop yields and food security. Moreover, the potential of fungal endophytes to enhance soil health and nutrient cycling holds promise for sustainable agriculture in the long term. With ongoing research and innovation, the integration of fungal endophytes into agricultural practices is likely to play a pivotal role in shaping a more sustainable and resilient future for global food production. Future research is required for better understanding of antagonistic behavior of these endophytic fungi to propagate them as environmentally friendly tools for managing phytopathogens naturally.

## Author contributions

MB: Writing – original draft. SKa: Writing – original draft. RK: Writing – original draft. SKh: Conceptualization, Writing – original draft, Writing – review & editing. SR: Supervision, Writing – review & editing.
